# The distinct effects of metabolic syndrome on negative symptoms and on antipsychotic therapy of schizophrenia involve insular volume, functional connectivity, and genetic polymorphisms

**DOI:** 10.1038/s41398-026-04167-3

**Published:** 2026-07-01

**Authors:** Jingyu Zhou, Mingjun Duan, Sisi Jiang, Huan Huang, Fei Wen, Yuling Luo, Yingjie Tang, Hui He, Qizhong Yi, María Luisa Bringas Vega, Lang Zhang, Gang Yao, Benjamin Klugah-Brown, Dezhong Yao, Cheng Luo

**Affiliations:** 1 The Clinical Hospital of Chengdu Brain Science Institute, MOE Key Lab for Neuroinformation, School of Life Sciences and Technology, University of Electronic Science and Technology of China, Chengdu, 610056 China; 2 Research Unit of Neuroinformation (2019RU035), Chinese Academy of Medical Sciences, Chengdu, 610072 China; 3 China-Cuba Belt and Road Joint Laboratory on Neurotechnology and Brain-Apparatus Communication, University of Electronic Science and Technology of China, Chengdu, 610054 P. R. China; 4 Department of Psychiatry, The Clinical Hospital of Chengdu Brain Science Institute, University of Electronic Science and Technology of China, Chengdu, 610056 China; 5 Psychological Medicine Center, The First Affiliated Hospital of Xinjiang Medical University, Urumqi, PR China; 6 Xinjiang Clinical Research Center for Mental Health, Urumqi, PR China; 7 Cuban Neuroscience Center, La Habana, Cuba; 8 Department of Radiology, The Fourth People’s Hospital of Chengdu, Chengdu, 610056 PR China

**Keywords:** Schizophrenia, Pathogenesis

## Abstract

As a prevalent comorbidity in schizophrenia, metabolic syndrome (MetS) exerts complex influences on residual symptoms and therapeutic outcomes of antipsychotic treatment. This study investigates the underlying neuroimaging, physiological and genetic mechanisms. At baseline, 142 participants were categorized into four groups according to diagnoses of schizophrenia and MetS. During follow-up, schizophrenia patients with and without MetS (SCZ-MetS and SCZ-nMetS) underwent 4-week antipsychotic treatment. Functional and structural magnetic resonance images, metabolic indicators and clinical scales were collected to investigate effects of MetS on gray matter volume (GMV), functional connectivity (FC) of schizophrenia patients, and related clinical implications. Using cytochrome P450 2D6 (CYP2D6) polymorphisms and brain-wide gene expression profiles, we explored genetic mechanisms of the therapeutic link between MetS and schizophrenia. SCZ-MetS showed greater insular GMV atrophy and functional dysconnectivity. Indirect effects of fasting blood glucose (FBG) on negative symptoms via insular GMV atrophy were observed. However, during follow-up, we observed group × time interactions on GMV and FC in insula. Increased GMV of the right insula in SCZ-MetS was correlated with the remission rate of positive symptoms. A moderation effect of FBG on this correlation was revealed. Finally, CYP2D6 polymorphisms and gene expression related to antipsychotic-response explained group × time interaction of GMV. Collectively, glucose metabolism, insular volume and FC underlie two distinct effects of MetS on: residual negative symptoms and therapeutic outcomes of positive symptoms in schizophrenia. The genetic mechanisms linking MetS to therapeutic outcomes involve CYP2D6 polymorphisms and the expression of genes related to antipsychotic-response.

## Introduction

Schizophrenia is regarded as one of the most serious psychiatric disorders with division of symptoms into positive symptoms, negative symptoms, and cognitive impairments [1]. Even when the antipsychotic treatment effectively alleviates positive symptoms, many patients suffer residual negative symptoms and cognitive deficits during the chronic phase of the illness. Additionally, patients with schizophrenia exhibit a higher prevalence of metabolic syndrome (MetS) than healthy individuals, particularly among those with chronic illness and a longer duration [2, 3]. Recent studies have revealed that peripheral metabolic dysregulation emerges as a risk factor for negative symptoms and cognitive impairments in patients with schizophrenia [4–6], suggesting a potential interplay between MetS and the residual symptoms of schizophrenia. However, the mechanisms linking metabolic dysregulation to the residual symptoms of schizophrenia are not well understood.

In neuroscience, the bidirectional interplay between the brain and peripheral metabolic systems is pivotal for the development and function of the nervous systems [7]. Dysfunction of peripheral metabolic systems, particularly glucose disturbances, adversely affects brain structure and function [8, 9]. Brain-apparatus communication with multiple physiological systems also emphasizes the critical role of metabolic system status in cognitive processes and psychiatric disorders [10]. Several studies have linked abnormal peripheral glucose metabolism to white matter disruption, suggesting a potential pathogenic mechanism of schizophrenia [11, 12]. Gray matter volume (GMV) loss, a prominent hallmark of schizophrenia that progressively worsens with longer illness duration or aging [13], may be further accelerated by the comorbidity of MetS, particularly in reward-related brain regions [14]. Additionally, neurostructural changes may lead to dysfunctions in circuits [15]. Functional connectivity (FC) alterations in cerebral reward circuit associated with peripheral metabolic function imply salient sensitization of dopaminergic system in schizophrenia patients with comorbidity of MetS [16]. Although the previous findings have indicated that comorbidity of MetS may exacerbate or precipitate structural and functional impairments in schizophrenia [17, 18], the relationship between these impairments and residual symptoms of schizophrenia remains unclear. Moreover, whether the structural and functional compromises in schizophrenia with comorbidity of MetS stem solely from MetS or result from the synergistic interaction between MetS and schizophrenia remains to be clarified through two-factor experimental design.

Under the prevailing view, MetS contributes to the morbidity, mortality, and cognitive impairment of patients with schizophrenia. However, potential beneficial effects of MetS on therapeutic outcomes of schizophrenia have also been reported and reviewed [19, 20]. Several studies have reported that physiological indices of MetS, such as body mass index [21], predict improvements in global psychopathology in schizophrenia [22, 23]. A systematic review and network meta-analysis including 25,952 schizophrenia patients supports the existence of an intrinsic therapeutic link between symptom improvement and metabolic dysregulation [24]. Neuroimaging and genetic mechanisms of this intrinsic therapeutic link between MetS and schizophrenia are expected to be further clarified through longitudinal experimental design [24].

Accumulating evidence supports that cytochrome P450 2D6 (CYP2D6) polymorphisms collectively contribute to inter-individual variations in antipsychotics response, metabolic adverse effects, and the susceptibility to schizophrenia [25, 26]. Therefore, CYP2D6 represents a key entry point for investigating genetic mechanisms underlying the relationship between treatment of schizophrenia and MetS. In this study, it is hypothesized that CYP2D6 polymorphisms and the expression profiles of genes involved in antipsychotic-response represent the genetic mechanisms underlying the intrinsic therapeutic link between MetS and schizophrenia.

The present study employed a two-factor design with longitudinal follow-up and integrated multimodal data, including neuroimaging, genetic, and peripheral metabolic measures, to examine the potential mechanisms underlying the effects of MetS on residual negative symptoms and short-term treatment response in schizophrenia. At baseline, we used two factors—Factor 1: diagnosis of schizophrenia and Factor 2: diagnosis of MetS—to categorize cross-sectional sample into four groups, including schizophrenia patients with comorbidity of MetS (SCZ-MetS), schizophrenia patients without MetS (SCZ-nMetS), healthy controls with MetS (HC-MetS) and healthy controls without MetS (HC-nMetS). During follow-up, SCZ-MetS and SCZ-nMetS received a 4-week antipsychotics treatment. At baseline, we examined the effects of MetS on schizophrenia symptoms and explored relevant mechanisms by analyzing the effects of MetS on the GMV and FC in patients with schizophrenia. In addition, the Factor 1×Factor 2 interaction effect on GMV and FC was examined at baseline. During follow-up, the group × time interaction on Positive and Negative Syndrome Scale (PANSS) scores was examined to determine therapeutic link between MetS and schizophrenia. Subsequently, the group × time interaction effect on GMV and FC was examined to identify potential neuroimaging mechanisms of the therapeutic link. Finally, we explored potential genetic mechanisms of the therapeutic link by analyzing CYP2D6 polymorphisms and expression profiles of the genes related to antipsychotic-response.

## Methods

### Participants

**Fig. 1:**
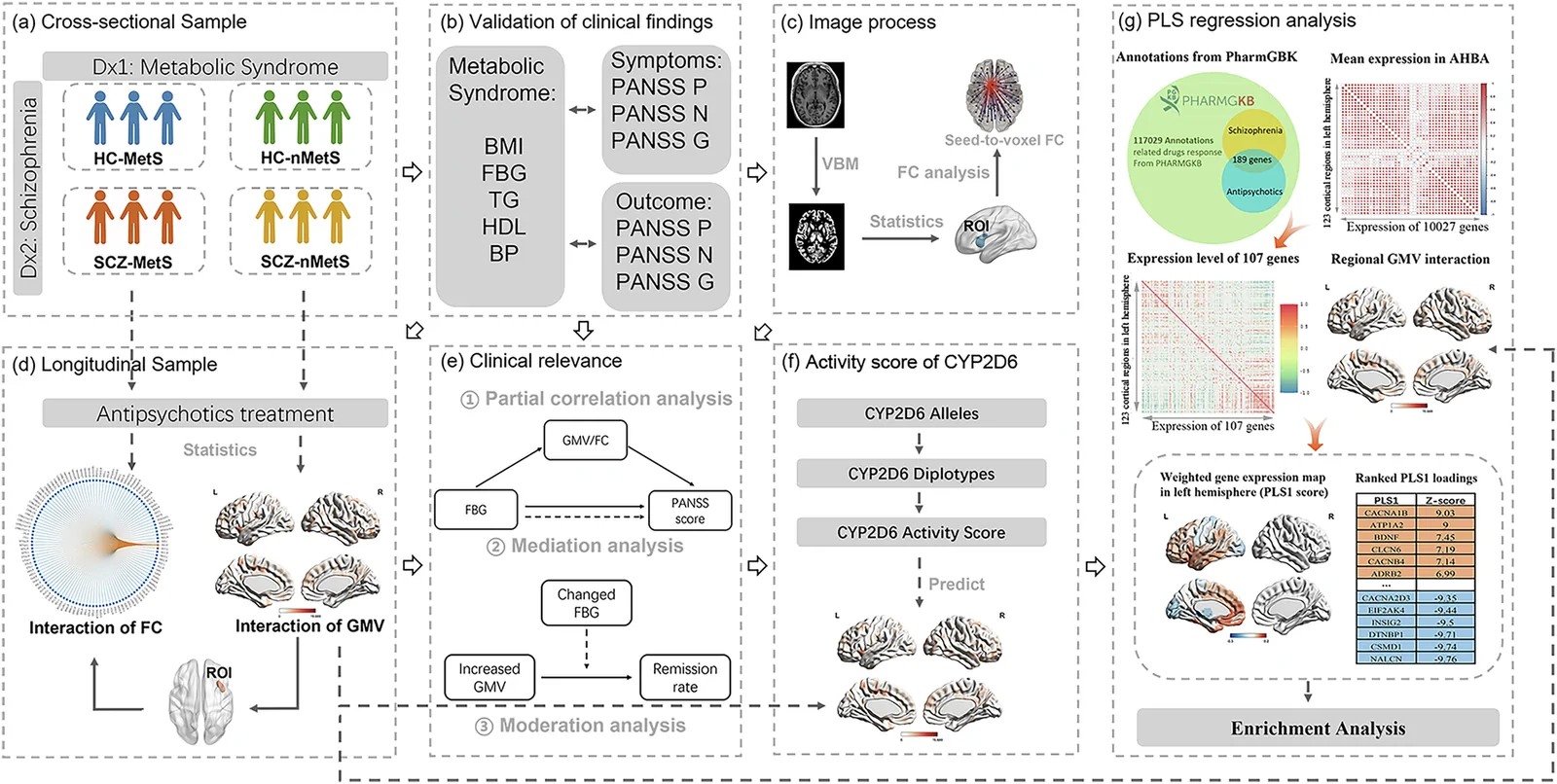
Work flow of the study. **a** Cross-sectional sample including schizophrenia patients with metabolic syndrome (SCZ-MetS), schizophrenia patients without metabolic syndrome (SCZ-nMetS), healthy controls with metabolic syndrome (HC-MetS) and healthy controls without metabolic syndrome (HC-nMetS) in two-factor experimental design. **b** Validation of clinical findings related to the impact of metabolic syndrome on symptoms and therapeutic outcomes of schizophrenia. **c** Image process, including voxel-based morphometry analysis and seed-to-voxel functional connectivity analyses. **d** A longitudinal sample of two groups of schizophrenia patients (SCZ-MetS and SCZ-nMetS) experiencing a 4-week antipsychotics treatment. **e** Examining clinical relevance of structural and functional changes at baseline and during the follow-up. **f** Individual cytochrome P450 2D6 (CYP2D6) activity score was used to predict the group × time GMV interaction patterns. **g** A hypothesis-driven partial least squares (PLS) regression was applied to explore imaging-transcriptomic associations between the group × time GMV interaction patterns and expression gradients of genes related to antipsychotic-response.

The experimental design and work flow of this study are summarized in Fig. 1. A total of 142 subjects were included, including 31 SCZ-MetS, 41 SCZ-nMetS, 39 HC-MetS, and 31 HC-nMetS. The sample size was determined using power analysis implemented in G*Power [27]. All the schizophrenia patients were recruited from the Clinical Hospital of Chengdu Brain Science Institute. The patients with schizophrenia were diagnosed using the Structured Clinical Interview according to the Diagnosis and Statistic Manual of Mental Disorders, fourth edition (DSM-IV) axis 1 disorders-clinical version (SCID-I-CV). This study employed a double-blind design. At both baseline and the follow-up assessment, the severity of positive and negative symptoms was evaluated by experienced psychiatrists who were blinded to group allocation using PANSS. The patients were also unaware of their group assignment. Additionally, healthy controls matched for age, gender, and education level were recruited (see Supplementary Methods 1).

For all participants, indicators of metabolic syndrome were measured, including BMI, fasting blood glucose (FBG), blood pressure (BP), triglyceride (TG), and high-density lipoprotein (HDL). Metabolic syndrome was diagnosed according to a slightly modified criterion based on the International Diabetes Federation in 2005 [28–30] (see Supplementary Methods 2). After a complete description of the study was given to the all participants and/or their guardians, written informed consent was obtained. The Ethics Committee of the Clinical Hospital of Chengdu Brain Science Institute in accordance with the Helsinki Declaration approved this study. Trial registration (Registration number: ChiCTR2100049273).

### Imaging acquisition and analysis

All MRI data were acquired on a 3 T MRI scanner (GE DISCOVERY MR750, USA) in the Center of Information Medicine Research in University of Electronic Science and Technology of China. High-spatial-resolution T1-weighted images were acquired by using a three-dimensional fast spoiled gradient-echo sequence. Resting-state brain functional magnetic resonance imaging (fMRI) was collected using gradient-echo echo-planar imaging (EPI) sequences. The main acquisition parameters were listed in Supplementary Methods 3.

High-spatial-resolution T1-weighted MRI data were processed with standard voxel-based morphometry (VBM) using the Computational Anatomy Toolbox (CAT12; http://dbm.neuro.unijena.de/cat12/) in statistical parametric mapping software (SPM12; http://www.fil.ion.ucl.ac.uk/spm/). The generated gray matter images were used as the GMV for subsequent group-level comparisons.

Functional imaging data were preprocessed using DPABI [31] and NIT toolboxes [32]. At baseline, regions exhibiting between-group differences in the GMV analysis were defined as regions of interest (ROIs) based on Automated Anatomical Labeling (AAL) atlas [33]. Subsequently, we performed seed-to-voxel whole-brain resting-state FC analyses for these ROIs. Similarly, during follow-up, regions exhibiting a group × time interaction effect on GMV were designated as ROIs to compute seed-to-voxel FC. The preprocessed blood oxygen level dependence (BOLD) time series were extracted from defined ROIs. Seed-to-voxel FC maps were generated by calculating Pearson’s correlations. Detailed steps of VBM analysis and preprocessing of resting-state fMRI are provided in Supplementary Methods 4 and 5.

### Genotyping of polymorphisms of CYP2D6 gene

Genotyping was performed to analyze the CYP2D6*39, CYP2D6*34, CYP2D6*2, CYP2D6*119 single nucleotide polymorphisms (SNP), and copy number variants (CNV) of CYP2D6 gene (see Supplementary Methods 6). Combining the alleles defined by measured SNP and CNV, the CYP2D6 diplotypes were classified as the phenotypes of normal metabolizer (NM) and ultrarapid metabolizer (UM) according to the expected enzyme activities reported on the database of the Pharmacogene Variation Consortium (PharmVar; https://www.pharmvar.org/gene/CYP2D6) [25]. Based on the clinical Pharmacogenetics Implementation Consortium guidelines for CYP2D6 genotype and codeine therapy, a CYP2D6 activity score was assigned to each diplotype as follows: 2 for NM; 3 or 4 for UM. The detailed schema of calculating individual CYP2D6 activity score is demonstrated in Supplementary Methods 7.

### Statistical analysis

First, the demographics and clinical characteristics were analyzed using SPSS version 26.0. The normality of continuous variables was assessed using the Shapiro-Wilk test. Homogeneity of variance for group comparisons was verified using Levene’s test. Differences in age and education level among the SCZ-MetS, SCZ-nMetS, HC-MetS, and HC-nMetS groups were assessed by one-way analysis of covariance (ANCOVA). Differences in gender were evaluated with the chi-square test. Comparisons between the SCZ-MetS and SCZ-nMetS groups were performed using two-sample t-tests for disease duration, medicine dose, and PANSS scores, and a Mann-Whitney U test for the CYP2D6 activity score. Second, one-way ANCOVA was performed to examine between-group differences of GMV and FC across the four groups. Based on the spatial location of difference from one-way ANCOVA, pairwise post-hoc comparisons were conducted using two-sample t-tests. Additionally, a two-way ANCOVA was performed to examine interaction effect of schizophrenia and MetS on GMV and FC at baseline. At baseline, all statistical analyses were performed with age, gender, education level and total intracranial volume (TIV) (for GMV analyses only) as covariates. Third, in the longitudinal analysis, a repeated measures ANCOVA was performed to examine changes in PANSS scores, indicators of MetS, GMV, and FC between the SCZ-MetS and SCZ-nMetS groups. This analysis yielded main effects of group, main effects of time, and group × time interaction effects. Post-hoc analysis was performed using paired t-tests to examine the differences between baseline and follow-up within each group. These longitudinal analyses controlled for age, gender, education level, total intracranial volume (TIV), and antipsychotic dosage.

### Relationship between clinical and neuroimaging features

In cross-sectional between-group comparisons, in order to explore the clinical relevance of the structural and functional alterations, we performed partial correlation analysis between clinical features—including PANSS scores and indicators of MetS—and neuroimaging features, including GMV and FC. In the correlation analyses, two-tailed tests were used.

In the longitudinal study, partial correlation analysis was used to quantify the relationship between remission ratio of the PANSS scores, metabolic change scores and neuroimaging features, including changes in GMV and FC. Furthermore, we also performed Spearman correlation analysis between the CYP2D6 activity score and the remission ratio of the PANSS scores, metabolic change scores, and changed neuroimaging features. Detailed methodologies for the extraction of neuroimaging features and the calculation of remission ratio of the PANSS scores and metabolic change scores are provided in Supplementary Methods 8.

Consistent with our previous study [23], we further evaluated the complex relationships among neuroimaging features, indicators of MetS, and PANSS scores using moderation model and mediation model implemented with Hayes’s PROCESS macro (Model 1 and Model 4) [34] in SPSS ver. 26.0.

### Partial least square (PLS) regression analysis

We selected a gene set associated with both “schizophrenia” and “antipsychotics” from 117,029 clinical annotations available in the Pharmacogenomics Knowledge Base (PharmGKB) [35]. By overlapping this gene set with the brain-wide gene expression data (only left hemisphere) of 10,027 genes obtained from Allen Human Brain Atlas (AHBA) [36], we obtained expression profiles for these genes.

PLS regression was used to analyze the relationship between the expression level of the gene set and the spatial pattern of the group × time interaction effect on GMV (see Supplementary Methods 9) [37–40]. Briefly, gene expression data were used as predictor variables of F-values of this GMV interaction effect in PLS regression. The first component of the PLS (PLS1) was identified as the linear combination of gene expression, which exhibited the strongest covariance with spatial pattern of the interaction effect on GMV.

### Enrichment analysis

A bioconductor package, the clusterProfiler 4.0 is designed to perform over-representation analysis (ORA) using gene ontology (GO) and Kyoto Encyclopedia of Gene and Genomes (KEGG) for diverse organisms. The analysis was carried out by R software (v.4.2.2) package clusterProfiler (v.4.5.0) [41] through Hiplot Pro (https://hiplot.com.cn/), a comprehensive web service for biomedical data analysis and visualization. We submitted all genes with significant weights on PLS1 to the package clusterProfiler and performed enrichment analysis based on three biological knowledge databases, including GO, KEGG, and disease ontology (DO). The significance of all enrichment pathways was thresholded at a false discovery rate (FDR) of 5%.

## Results

### Demographics and clinical characteristics

The demographic and clinical characteristics of the four participant groups are presented in Table 1. One patient with schizophrenia from the SCZ-nMetS group was excluded due to excessive head motion, yielding a final sample of 31 SCZ-MetS, 40 SCZ-nMetS, 39 HC-MetS, and 31 HC-nMetS participants for final analysis.

**Table 1:** Demographic information and clinical characteristics of four groups of participants at baseline (*n* = 141).

Characteristic	SCZ-MetS (*n* = 31)	SCZ-nMetS (*n* = 40)	HC-MetS (*n* = 39)	HC-nMetS (*n* = 31)	Unadjusted
	Mean (SD)	Mean (SD)	Mean (SD)	Mean (SD)	*p*-value
Gender (male/female)	26/5	27/13	29/10	28/3	0.11
Age (years)	45.16 (8.8)	40.07 (12.13)	43.64 (9.98)	42.26 (10.27)	0.26
Education (years)	12.1 (2.68)	11.53 (2.65)	11.67 (4.06)	11.95 (3.66)	0.94
Disease duration (years)	20.55 (9.18)	16.61 (11.54)	–	–	0.12
Medicine dose (chlorpromazine equivalent)	338.04 (162.75)	321.13 (134.2)	–	–	0.59
PANSS total score	62.79 (14.11)	61.8 (12.32)	–	–	0.78
PANSS positive symptoms score	11.5 (5.11)	14.87 (5.75)	–	–	0.02
PANSS negative symptoms score	22.54 (6.37)	19.77 (5.94)	–	–	0.09
PANSS general symptoms score	28.75 (5.99)	27.17 (5.37)	–	–	0.29

At baseline, we did not collect complete clinical data for 71 patients. Only 54 patients’ PANSS scales and 64 patients’ BMI were collected at baseline.

No significant differences in gender, age, or education level were observed among the four groups. In addition, no significant difference in medication dosage was observed between the SCZ-MetS and SCZ-nMetS groups. At baseline, we only collected PANSS scales of 54 patients. Primary clinical relevance analysis at baseline was conducted on these 54 patients. No differences were observed in negative symptoms and general symptoms (Table 1). However, we found that the SCZ-MetS group had lower positive symptom scores than the SCZ-nMetS group (*t* = −2.35, *p* < 0.05). The metabolic characteristics of the participants are presented in Table S1 and Table S2.

After the 4-week treatment, 33 patients with schizophrenia (17 SCZ-MetS and 16 SCZ-nMetS) completed the follow-up and constituted the longitudinal analysis cohort. Their clinical characteristics are summarized in Table S3 and Table S4. Longitudinal analysis revealed a significant main effect of time on all PANSS symptom domains. However, we did not find significant group × time interaction on the PANSS scores. We did not find significant group × time interaction effects or main effects of time on metabolic indexes (Table S4).

### Between-group difference of GMV and clinical relevance at baseline

**Fig. 2:**
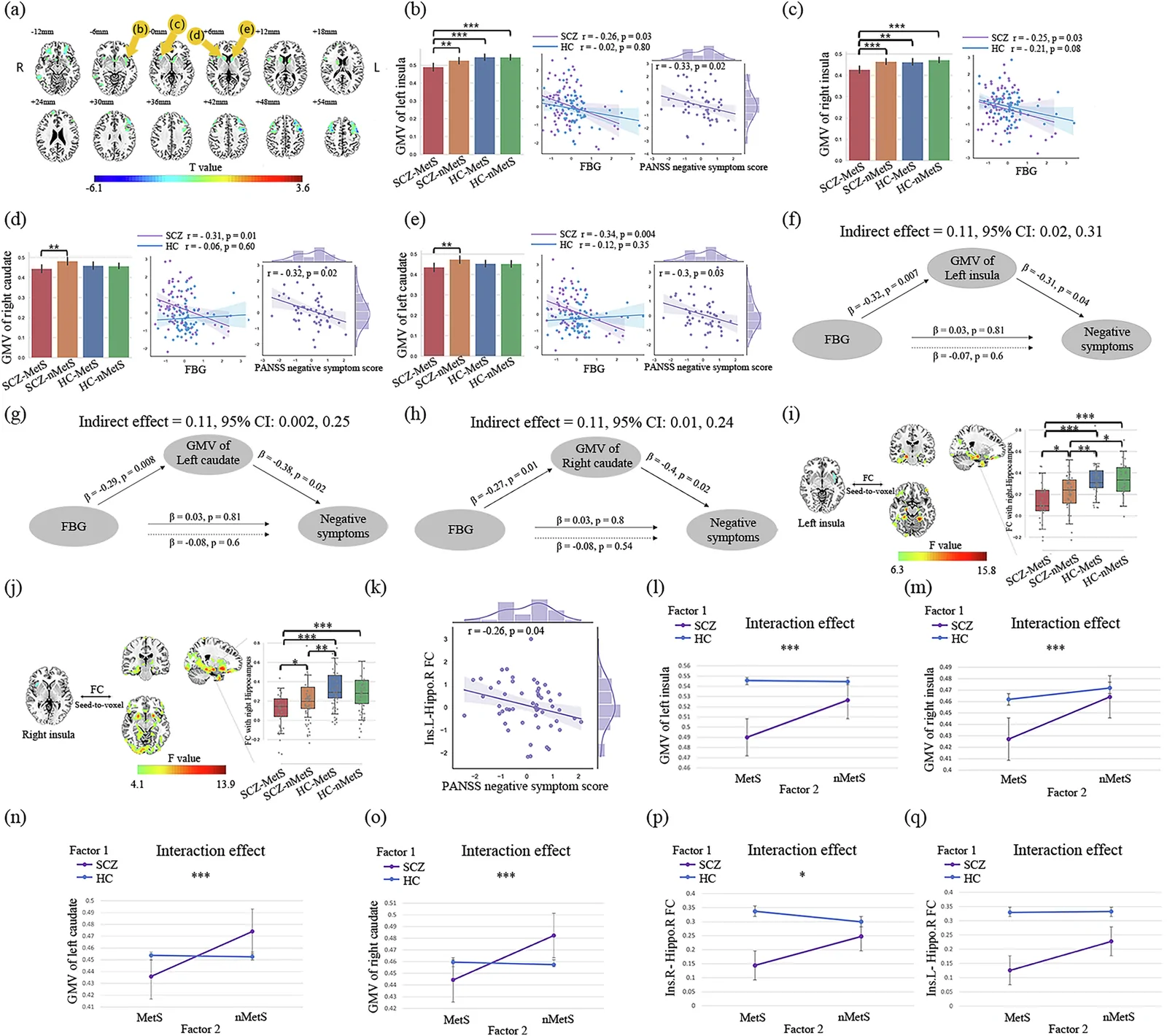
Group difference of GMV and FC, and their clinical relevance at baseline. **a** Map of t-statistic showing GMV differences between SCZ-MetS and SCZ-nMetS (*p* < 0.05, FDR corrected); (**b**–**e**) Left bar plots show the GMV differences in the bilateral insula and bilateral caudate among the four groups (SCZ-MetS, SCZ-nMetS, HC-MetS, HC-nMetS); Middle scatter plots show the association of GMV in corresponding regions with peripheral fasting blood glucose, in purple for schizophrenia group (SCZ-MetS and SCZ-nMetS) and in blue for non-schizophrenia group (HC-MetS and HC-nMetS); Right scatter plots show association of GMV in corresponding regions with PANSS negative symptom scores in schizophrenia group. **f**–**h** The indirect effect of FBG on negative symptom via GMV of the left insula and bilateral caudate in schizophrenia group. **i,** **j** Left map: mask of GMV atrophy in bilateral insula. Middle map: map of F-statistic values for one-way ANCOVA of bilateral insula seed-to-voxel FC. Right box plot: The differences of FC between bilateral insula and right hippocampus. **k** Scatter plot shows correlation of FC between left insula and right hippocampus with negative symptom score in schizophrenia group. **l**–**o** The Factor 1×Factor 2 interaction effect of GMV in bilateral insula and bilateral caudate (*p* < 0.05, FDR corrected, error bars represent standard error of the mean (SEM)). **p,** **q** The Factor 1×Factor 2 interaction effect of FC between bilateral insula and right hippocampus (*p* < 0.05, uncorrected, error bars represent SEM). Ins.L-Hippo.R FC: FC between left insula and right hippocampus; Ins.R-Hippo.R FC: FC between right insula and right hippocampus; *p < *0.05*, **p < *0.01*, ***p < *0.001*.

One-way ANCOVA revealed GMV differences among the four groups (Figure S1). Post-hoc analyses indicated that the SCZ-MetS group showed reduced GMV in the bilateral insula compared with the HC-MetS and HC-nMetS groups (Fig. 2b, c). Furthermore, the SCZ-MetS group showed more pronounced GMV atrophy in the bilateral insula compared with the SCZ-nMetS group (Fig. 2a, *p* < 0.05, FDR corrected; Fig. 2b, c). While the SCZ-MetS group did not show significant difference in GMV within the bilateral caudate compared with healthy controls, they exhibited a GMV reduction in this region compared to the SCZ-nMetS group (Fig. 2a, *p* < 0.05, FDR corrected; Fig. 2d, e). Importantly, no significant GMV differences in these regions were observed between the HC-MetS and HC-nMetS groups. The results of the post-hoc analyses for other brain regions are shown in Figure S2.

In the schizophrenia group (SCZ-MetS and SCZ-nMetS groups), we revealed negative correlations between peripheral FBG levels and GMV in the bilateral insula (left: *r* = −0.26, *p* = 0.03 in Fig. 2b; right: *r* = −0.25, *p* = 0.03 in Fig. 2c) and bilateral caudate (right: *r* = −0.31, *p* = 0.01 in Fig. 2d; left: *r* = −0.34, *p* = 0.004 in Fig. 2e, FDR corrected). However, these correlations were not observed in non-schizophrenia group (HC-MetS and HC-nMetS groups). Additionally, we found negative correlations between PANSS negative symptom scores and GMV in left insula (*r* = −0.33, *p* = 0.02 in Fig. 2b) and bilateral caudate (right: *r* = −0.32, *p* = 0.02 in Fig. 2d; left: *r* = −0.3, *p* = 0.03 in Fig. 2e) in schizophrenia group.

Mediation analysis respectively revealed indirect effect of FBG on negative symptoms via GMV reduction in three regions, including the left insula (*indirect effect* = 0.11, 95% *CI* 0.02 to 0.31, in Fig. 2f) and bilateral caudate (left: *indirect effect* = 0.11, 95% *CI* 0.002 to 0.25, in Fig. 2g; right: *indirect effect* = 0.11, 95% *CI* 0.01 to 0.24, in Fig. 2h). However, the total and direct effects of FBG on negative symptoms were not significant.

### Between-group difference of FC and clinical relevance at baseline

At baseline, we selected the atrophic bilateral insula and caudate as ROIs to compute seed-to-voxel FC. One-way ANCOVA revealed the between-group differences in FC among 4 groups (Figure S3, *p* < 0.05, FDR corrected). Compared with the HC-MetS and HC-nMetS groups, the SCZ-MetS group exhibited reduced FC between the bilateral insula and right hippocampus, while the SCZ-nMetS group only showed reduced FC between left insula and right hippocampus (Fig. 2i, j). Compared with the SCZ-nMetS group, the SCZ-MetS group showed more pronounced FC reductions between the bilateral insula and right hippocampus.

We found that FC between left insula and right hippocampus was negatively correlated with negative symptom scores (Fig. 2k; *r* = −0.26, *p* = 0.04). No significant correlation between FC and FBG level was observed. Furthermore, no significant indirect effect of FC was observed.

### Interaction of factor 1 and factor 2 on GMV, FC at baseline

At baseline, two-way ANCOVA demonstrated interaction effects between schizophrenia and MetS on GMV in the bilateral insula and caudate (Fig. 2l–o, *p* < 0.05, FDR corrected). In addition, an interaction effect between schizophrenia and MetS on FC between the right insula and right hippocampus was revealed (Fig. 2p, *p* < 0.05, uncorrected). However, no significant interaction effect on FC was observed between the left insula and right hippocampus (Fig. 2q). No significant interaction effect on insular FC was observed after FDR correction (Fig. 2p, q).

### Interaction of group and time on GMV and clinical relevance during the treatment

**Fig. 3:**
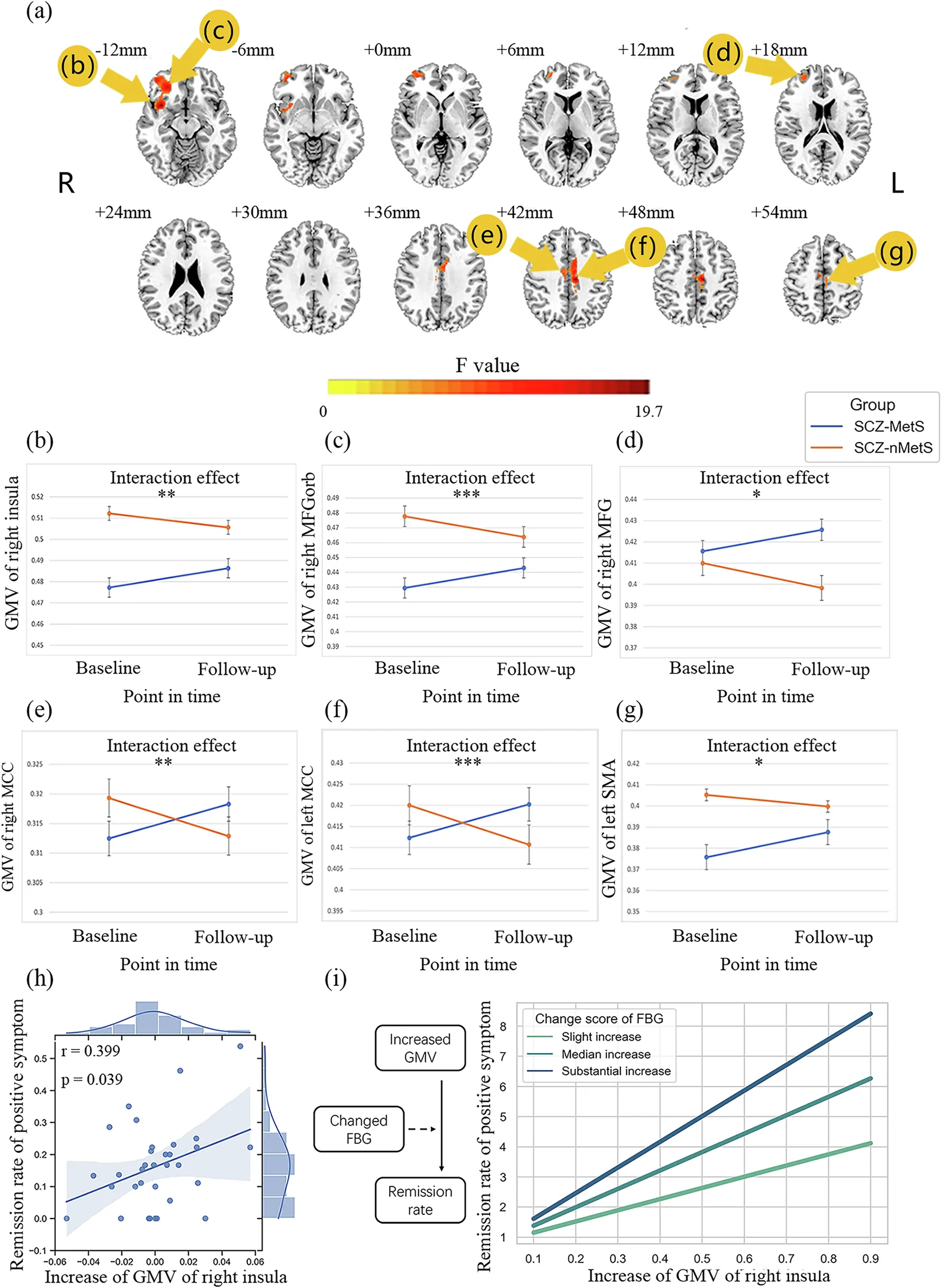
Group × time interaction effect of GMV and clinical relevance in longitudinal study. **a** Map of F-statistic values for repeated measures ANCOVA showed the group × time interaction effect on the GMV (*p* < 0.05, GRF-corrected). **b** The group × time interaction effect of GMV within right insula. **c** The group × time interaction effect of GMV within right orbital middle frontal gyrus. **d** The group × time interaction effect of GMV within right middle frontal gyrus. **e** The group × time interaction effect of GMV within right middle cingulum cortex. **f** The group × time interaction effect of GMV within left middle cingulum cortex. **g** The group × time interaction effect of GMV within left supplementary motor area. **h** Scatter plot showing a positive correlation between the increased GMV within right insula and the remission rate of positive symptoms. **i** The moderation effect of change scores of FBG on the correlation between the changed GMV of right insula and the remission rate of positive symptom. MFGorb: orbital middle frontal gyrus; MFG: middle frontal gyrus; MCC: middle cingulum cortex; SMA: supplementary motor area. *p < *0.05*, **p < *0.01*, ***p < *0.001*.

Repeated measures ANCOVA revealed group × time interaction effects on GMV within the right insula, right orbital middle frontal gyrus, right middle frontal gyrus, bilateral middle cingulum cortex (MCC) and left supplementary motor area (SMA) (Fig. 3a, *p* < 0.05, Gaussian Random Field (GRF)-corrected). Although no significant interaction effect remained after FDR correction, we revealed the presence of these interaction effects with large effect sizes (*partial η*^*2*^ = 0.2–0.5). Post-hoc analyses revealed that the SCZ-MetS group exhibited increased GMV in these brain areas showing a significant interaction, while the SCZ-nMetS group presented reduced GMV in these regions (Fig. 3b–g).

Partial correlation analysis revealed that increased GMV in the right insula was positively correlated with the remission rate of positive symptoms (Fig. 3h, r = 0.39, *p* = 0.039). Furthermore, this correlation was moderated by the change scores of FBG (Fig. 3i, Table S5).

### Interaction of group and time on FC and clinical relevance during the treatment

During the follow-up, we defined ROIs based on the brain regions showing a group × time interaction on GMV and performed seed-to-voxel FC analysis. Repeated measures ANCOVA revealed the group × time interaction effects on the FC of these ROIs (Figure S4, *p* < 0.05, GRF-corrected). Although no significant interaction of FC remained after FDR correction, we revealed the presence of these interaction effects with large effect sizes (*partial η*^*2*^ = 0.26–0.4). Interestingly, we observed interaction effects on the FC between the right insula and several other regions with significant GMV interactions, including the bilateral MCC, left SMA, and right orbital middle frontal cortex (Figure S5a). Additionally, FC interaction effects were identified between the right insula and left cerebellum lobules Ⅷ as well as the right inferior parietal gyrus.

Post-hoc analysis revealed that the SCZ-MetS group showed increased FC between the right insula and the bilateral MCC and left SMA (Figure S5b-c, Figure S5e). The SCZ-nMetS group showed reduced FC between the right insula and the bilateral MCC and left cerebellum lobules Ⅷ (Figure S5b-d). Furthermore, the increased FC between the right insula and the SMA was correlated with a higher remission rate of PANSS total scores (Figure S5f, r = 0.39, *p* = 0.04).

### Relationship between activity score of CYP2D6 and increased GMV

**Fig. 4:**
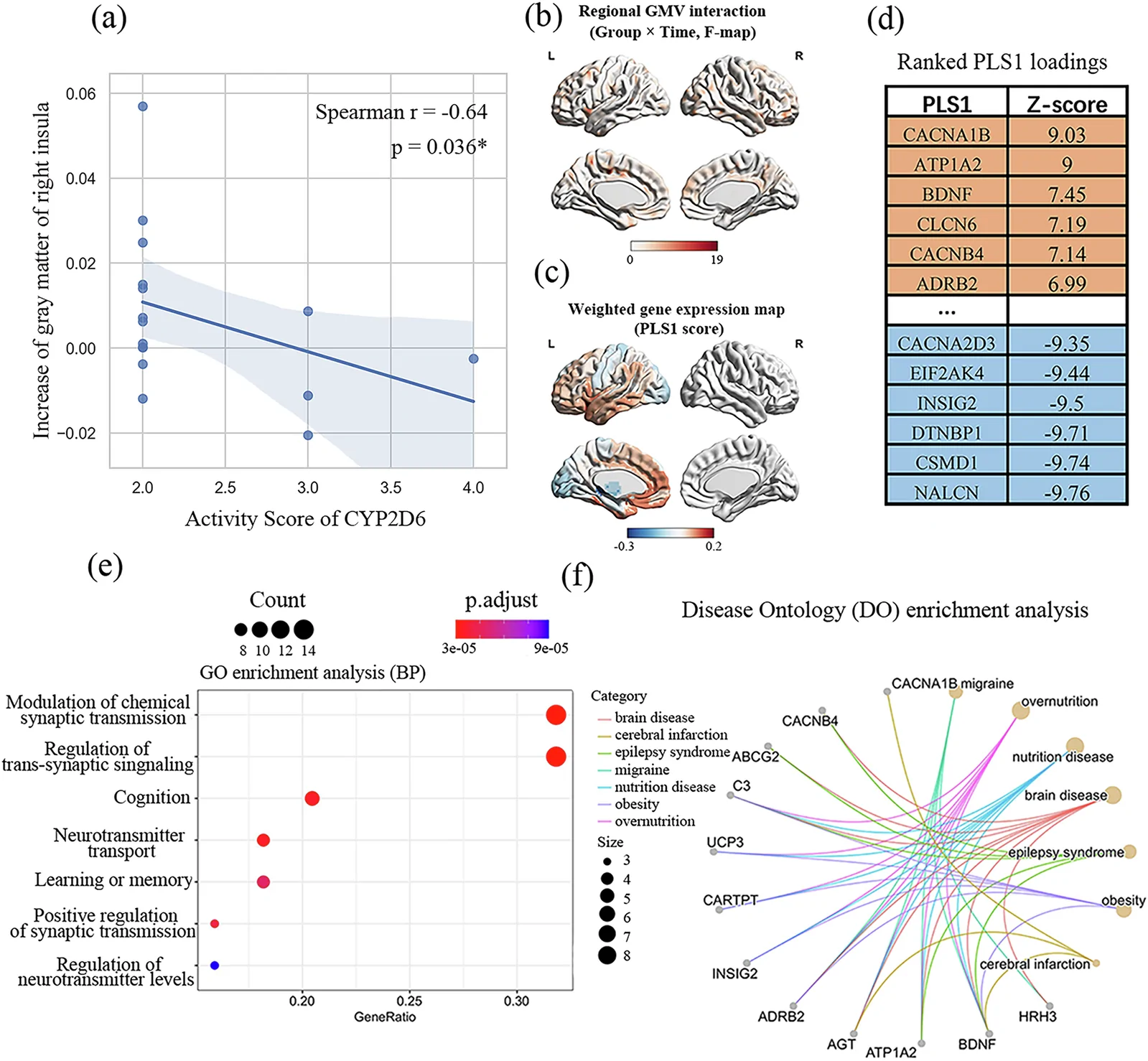
Relationships among CYP2D6 activity score, gene expression profiles and regional GMV interaction pattern. **a** Correlation between the CYP2D6 activity score and the longitudinal increase in GMV within the right insula; (**b**) Statistical F-map of the group × time interaction effect on GMV (unthresholded); (**c**) A weighted gene expression map of regional PLS1 scores in the left hemisphere (unthresholded); (**d**) Ranked PLS1 loadings of top six PLS1^+^ genes and top six PLS1^−^ genes; (**e**) Enrichment analysis based on gene ontology (GO) biological processes displaying the top seven enriched terms (*p* < 0.05, FDR corrected); (**f**) Enrichment analysis based on disease ontology (DO) displaying the top seven enriched terms related to disease (*p* < 0.05, FDR corrected).

Based on the polymorphisms and CNV of the CYP2D6 gene, CYP2D6 phenotypes of all 16 patients were classified into NM (*n* = 12) and UM (*n* = 4). CYP2D6 activity score were assigned to each diplotype as follows: 2 for NM; 3 or 4 for UM. We found a negative correlation between the CYP2D6 activity score and longitudinal increase in GMV in the right insula (Fig. 4a, r = −0.64, *p* = 0.036, *n* = 16).

### Relationship between group × time GMV interaction pattern and gene expression profile

From the 117,029 annotations of PharmGKB, we selected 189 genes related to both “schizophrenia” and “antipsychotics”. By intersecting this gene set with brain-wide expression data of 10,027 genes, we derived the expression profiles of 107 candidate genes.

PLS1 was selected as the spatial map that explained the 107 genes’ expression variance across cortical areas to the maximum extent (Fig. 4b, Fig. 4c). PLS1 explained 25% of the variance of GMV interaction pattern (*p* < 0.01, permutation test). The weights of PLS1 were normalized based on univariate one-sample Z tests. We ranked the weights and found 32 negative weights (PLS1^−^) genes and 12 positive weights (PLS1^+^) genes (Fig. 4d, *p* < 0.05, FDR corrected; Table S6).

### Enrichment pathways associated with group × time GMV interaction pattern

Enrichment analysis identified several significant enriched terms from the GO, KEGG and DO databases. (Fig. 4e, f, Figure S6, *p* < 0.05, FDR corrected). The top seven GO biological processes were primarily involved in synaptic function, cognitive function, and neurotransmitter transmission (Fig. 4e). GO molecular functions were enriched in “voltage-gated channel activity” (Figure S6a), while cellular components were associated with “ion channel complex” (Figure S6b). KEGG pathway analysis highlighted terms related to “cardiomyopathy” (Figure S6c). Furthermore, DO enrichment analysis revealed terms including “brain disease” and “obesity”. Genes “C3”, “ADRB2”, and “BDNF” were linked to both “brain disease” and “obesity” (Fig. 4f).

## Discussion

This study incorporated two-factor and longitudinal experimental designs and employed multimodal data to elucidate the mechanisms by which MetS influences negative symptoms and short-term therapeutic outcomes in schizophrenia. First, we demonstrated a more prominent impact of MetS on insular structure and function in patients with schizophrenia. The glucose disturbance may exacerbate negative symptoms by deteriorating the GMV atrophy of the insula and caudate. Second, during short-term treatment, group × time interaction effects on insular GMV and FC were revealed. In the SCZ-MetS group, distinct longitudinal trajectories of insular GMV were associated with the remission rate of positive symptoms, suggesting a potential mechanism underlying the influence of MetS on therapeutic outcomes in schizophrenia. Finally, the genetic basis of the intrinsic therapeutic link between MetS and schizophrenia involved individual genetic polymorphisms of CYP2D6 and global expression patterns of genes associated with antipsychotic-response. The enrichment analysis revealed potential ontological pathways implicated in this therapeutic link. This study provides longitudinal neuroimaging and genetic evidence suggesting that metabolic syndrome exerts two dissociable influences on schizophrenia: a baseline glucose-related detrimental effect on insular–striatal circuitry associated with negative symptoms, and a short-term treatment-related modulation of insular plasticity linked to improvement in positive symptoms.

First, our results indicate that MetS, particularly peripheral glucose disturbances, may exacerbate negative symptoms in schizophrenia by contributing to GMV atrophy within the reward circuit. A previous study has revealed that structural brain volume reductions in areas of the reward circuit appear to be related to comorbid MetS in schizophrenia [14]. The possible mechanisms linking MetS and negative symptoms, including insulin resistance and chronic elevation of FBG, are considered key contributors to GMV reduction [8, 42]. The current study reveals the potential mechanisms underlying the effect of glucose disturbances on negative symptoms of schizophrenia, which involve GMV atrophy of the insula and caudate. Additionally, in the SCZ-MetS group, further reduced FC between the atrophic left insula and the right hippocampus was correlated with negative symptom severity. These results suggest that the impact of glucose disturbances on negative symptoms may be involved in reward circuit dysfunction associated with brain structural changes. Shared dysfunction of D2 dopaminergic receptors in schizophrenia and MetS may also underlie the observed functional synchronization abnormalities [43–46]. However, some correlations between GMV, glucose disturbances, and symptoms in the current study did not survive correction for multiple comparisons. Thus, these findings should be validated in larger independent cohorts. In addition, as key regions in the reward circuit, structural alterations in the insula and caudate may promote poor health choices, potentially increasing risk of hyperglycemia. Further research is needed to determine the causality.

Previous studies have suggested that the effects of glucolipid metabolism on brain structure were more pronounced in schizophrenia patients than in the general population [47, 48]. Utilizing a two-factor experimental design, our study reveals a more prominent impact of MetS on GMV atrophy of the insula and caudate in schizophrenia. The specific negative correlations of GMV in the insula and caudate with FBG observed in the schizophrenia group further support our hypothesis. One plausible explanation is that shared pathophysiological mechanisms between MetS and schizophrenia—such as insulin resistance and inflammation—may heighten the vulnerability of individuals with schizophrenia to the impact of MetS risk factors on brain morphology.

Although previous clinical studies have documented the influence of MetS on antipsychotic efficacy, more objective endophenotype markers and underlying mechanisms are lacking [22, 24]. Our longitudinal analysis revealed group × time interaction effects, indicating divergent trajectories of GMV and FC changes between SCZ-MetS and SCZ-nMetS groups during the antipsychotic treatment. Insula played a central role in these interaction effects. In chronic patients, GMV atrophy is continuously progressive [49], but antipsychotic treatment may attenuate brain tissue reduction in frontal and insular regions [15, 50, 51]. Therefore, the correlation observed between increased insular GMV in the SCZ-MetS group and the remission rate of positive symptoms may reflect a better response to antipsychotics. The moderation effect of FBG on this correlation further indicate that patients with elevated FBG levels during treatment may exhibit enhanced therapeutic responses. This observation aligns with a systematic review elucidating the relationships between glucose parameters and clinical improvement in psychiatric disorders [52]. Collectively, these findings suggest that the emergence of MetS during antipsychotic treatment may modulate different trajectories of GMV and FC especially in the insula, which are associated with therapeutic outcomes. Our study provides novel insights into the potential mechanisms through which MetS beneficially impact the short-term therapeutic responses in schizophrenia.

The present results did not reveal divergent trajectories of caudate GMV changes during antipsychotic treatment. One possible explanation is the distinct functional roles of the caudate relative to the insula. The caudate nucleus, as a component of the striatum, is primarily involved in reward-based learning and motivational processing [53]. In contrast, the insula plays a key role in interoceptive awareness and is thought to be particularly sensitive to internal metabolic state of the body, including blood glucose levels and insulin signaling [54, 55]. Importantly, the previous studies suggested that the relationship between glucose dysregulation and clinical improvement might be related to insulin signaling pathway [52]. The relatively lower sensitivity of the caudate to insulin signaling pathways, together with its potentially slower structural plasticity, may partly explain the weaker longitudinal effects observed in this region. Nevertheless, as the caudate is considered as a primary target of efficacious antipsychotics, the potential role of the caudate nucleus in the potential mechanisms through which MetS influence therapeutic responses in schizophrenia warrants further investigation.

There are two distinct effects of MetS on schizophrenia [20, 24]. On one hand, MetS contributes to residual negative symptoms of schizophrenia [5, 56]. On the other hand, possible beneficial effects of MetS on therapeutic outcome in schizophrenia have been reported [22, 57]. Our findings reveal neurobiological mechanisms underlying the influences of MetS on residual negative symptoms and therapeutic outcomes in schizophrenia. Specifically, peripheral glucose disturbances may exacerbate negative symptoms by contributing to GMV atrophy in the insula and caudate. This may be a long-term detrimental effect. However, during short-term antipsychotic treatment, the positive moderation effect of FBG on the correlation between increased GMV in the right insula and the remission rate of positive symptoms indicates that emergence of MetS is associated with better therapeutic outcomes of positive symptoms. This may be a short-term beneficial effect. Thus, our findings show two distinct pathways through which MetS influences schizophrenia. The underlying mechanisms involve glucose metabolism and insular volume and functional connectivity.

In this study, we preliminarily explored possible genetic mechanisms underlying the intrinsic therapeutic link between MetS and schizophrenia treatment. First, we identified a correlation between the CYP2D6 activity score and increased GMV in the right insula during treatment. Furthermore, DO enrichment analysis revealed both “brain disease” and “obesity” terms. Our results suggest that certain genes implicated in both psychiatric disorders and obesity may help explain GMV interaction patterns underlying the intrinsic therapeutic link between MetS and schizophrenia treatment. A recent study reported similar findings that a subgroup of schizophrenia patients with enhanced therapeutic response and weight gain showed distinctive genetic features involving genes associated with obesity-related traits and psychosis [19]. Second, recent pharmacogenetics evidence has demonstrated that maintaining an optimal antipsychotic plasma concentration, largely determined by CYP2D6 polymorphisms, is critical for clinical response [58–60]. The CYP2D6-associated white matter alterations in the hippocampal have been linked to individual variability in treatment response [25]. Building on this foundation, our results suggest that individual CYP2D6 activity score help explain different GMV trajectories in right insula, which involve the potential mechanisms underlying the intrinsic therapeutic link. Finally, the relationship between cortical expression profiles of the 107 identified genes and GMV interaction pattern underscores the putative involvement of genes related to antipsychotic-response in the therapeutic link. Enrichment analysis indicated that these genes are primarily involved in synaptic transmission, voltage-gated channel activity, and ion channel complex. However, more specific biological pathways underlying this intrinsic therapeutic link require further detailed investigation.

### Limitations

There are still some limitations in our current research. First, the high dropout rate in our longitudinal study may introduce selection bias and limit the generalizability of the findings. Second, we did not consider use and dosage of lipid-lowering, hypoglycemic, or anti-hypertensives agents as confounding factors. These medications may blur metabolic state and influence brain GMV loss [61]. Third, in the longitudinal analysis, the group × time interaction effects did not remain significant after more stringent FDR correction, and no significant group × time interaction was found for the PANSS scores. These results may be limited by the small sample size. Finally, although some correlations between the GMV, glucose disturbances, symptoms, and CYP2D6 activity score were revealed, these results should be interpreted with caution due to the limited statistical power from the small sample size. Future studies should employ prospective longitudinal designs with larger sample sizes and encompassing diverse schizophrenia subgroups, such as patients experiencing weight gain without therapeutic response. In addition, it is crucial to account for a broader range of confounding factors, including medicine, dietary habits, and physical activities.

## Conclusion

In summary, glucose metabolism, insular volume, and functional connectivity underlie two distinct effects of MetS on schizophrenia. Our study identified unique neuroimaging characteristics of SCZ-MetS and highlighted the central role of the insula in potential mechanisms by which MetS influences residual negative symptoms and short-term treatment outcomes of positive symptoms in schizophrenia. We extend previous findings by demonstrating a more prominent impact of MetS on insular structure and function in schizophrenia. Furthermore, the genetic mechanisms of the intrinsic therapeutic link between MetS and schizophrenia involve individual CYP2D6 polymorphisms and global expression patterns of genes related to antipsychotic-response.

## Supplementary information

Supplementary Material

## Data Availability

The expression profiles of the genes from Allen Human Brain Atlas (AHBA) can be found at: https://human.brainmap.org/static/download. Clinical annotations from Pharmacogenomics Knowledge Base (PharmGKB) can be found at: https://www.pharmgkb.org/downloads. Other data and information supporting the findings of this study are available from the corresponding author upon reasonable request through e-mail: chengluo@uestc.edu.cn.

## Code availability

The code for PLS regression analysis used in this study can be found at https://github.com/jssuestc/MultimodalCorr.
