# Supplementary material for: The distinct effects of metabolic syndrome on negative symptoms and on antipsychotic therapy of schizophrenia involve insular volume, functional connectivity, and genetic polymorphisms
Source: Transl Psychiatry. 2026 Jul 1;16:476. doi: 10.1038/s41398-026-04167-3 (PMC13594184; doi:10.1038/s41398-026-04167-3)
Supplement: Supplementary file 1 [file 41398_2026_4167_MOESM1_ESM.docx]

Supplementary materials for “The Distinct Effects of Metabolic Syndrome on Negative Symptoms and on Antipsychotic Therapy of Schizophrenia Involve Insular Volume, Functional Connectivity, and Genetic Polymorphisms”.

**Supplementary Methods**

**1. Participant recruitment**

A total of 142 subjects were acquired in this study, including 31 schizophrenia patients with comorbidity of metabolic syndrome (SCZ-MetS), 41 schizophrenia patients without metabolic syndrome (SCZ-nMetS), 39 healthy controls with metabolic syndrome (HC-MetS), and 31 healthy controls without metabolic syndrome (HC-nMetS). All the schizophrenia patients were recruited from the Clinical Hospital of Chengdu Brain Science Institute. The patients were diagnosed using the Structured Clinical Interview according to the Diagnosis and Statistic Manual of Mental Disorders, fourth edition (DSM-IV) axis 1 disorders-clinical version (SCID-I-CV). All patients had been treated with antipsychotics. The illness duration of the patients was at least 2 years. The severity of positive and negative symptoms was evaluated by experienced psychiatrists using Positive and Negative Syndrome Scale (PANSS) within one week of magnetic resonance imaging (MRI) scanning. Schizophrenia patients were excluded from the study if they were diagnosed with organic mental disorders, other serious physical disease, psychosis associated with substance use, pervasive developmental disorder, a history of epilepsy, and alcohol dependence; were pregnant or breastfeeding; had contraindications for MRI scanning. Healthy controls (HC) were recruited from the local community through advertisements and word of mouth to participate in this study. HC were excluded based on current or past axis I disorder as verified using the Structure Clinical Interview for DSM-IV, history of neurological illness, traumatic brain injury, substance-related disorders, or first-degree relatives with history of psychosis. During the functional magnetic resonance imaging (fMRI) scanning, all participants whose maximum translation in any of the orthogonal directions larger than 2.5 mm or rotation larger than 2.5° were excluded from subsequent analysis. One patient was excluded due to exceeded head motion. Thirty-one SCZ-MetS (45.16 ± 8.8 years old), 40 SCZ-nMetS (40.07 ± 12.13 years old), 39 HC-MetS (43.64 ± 9.98 years old) and 31 HC-nMetS (42.26 ± 10.27 years old) were included in the final analysis.

Longitudinal sample was a subsample of aforementioned cross-sectional sample. All patients with schizophrenia in longitudinal sample received 4 weeks’ antipsychotics treatment.

**2. Assessments of metabolic syndrome**

All indicators used for the diagnosis of metabolic syndrome were measured, including height, weight, fast blood glucose (FBG), blood pressure (BP), [triglyceride](javascript:;) (TG), and high-density lipoprotein (HDL). The metabolic syndrome history of patients with schizophrenia were found in their medical histories. The metabolic syndrome history of two groups of healthy controls were typically obtained through interviews. Body mass index (BMI) was calculated as the participant's body weight in kilograms (kg) divided by his height in meters squared (m^2^) ^−1^. Systolic blood pressures (SBP) and diastolic blood pressures (DBP) were assessed by a digital blood pressure monitor. Peripheral venous blood samples were collected from participants. The FBG level [test](javascript:;) and lipid profiles were performed for each participant.

For the diagnose of metabolic syndrome in this study, considering Asians’ ethnic specificity of the relationship between BMI and body fat percentage (1), we lowered the BMI cut-off value in this study, from 30kg m^−2^ to 27kg m^−2^. Besides that, due to standpoint that central obesity should be an "optional" rather than "essential" criterion for defining metabolic syndrome in Asian cohort, whether a participant was diagnosed with metabolic syndrome was determined according to a slightly modified criteria based on International Diabetes Federation in 2005 (2, 3). Participants were diagnosed with metabolic syndrome if they displayed random three of the following criteria defined by International Diabetes Federation: central obesity (BMI ≥27 kg/m^2^); (2) FBG ≥5.6 mmol/L or treatment for diabetes mellitus; (3) BP ≥130/85 mmHg or on anti-hypertensives; (4) TG ≥1.695 mmol/L or on lipid lowering agent; (5) HDL＜1.036 mmol/L for men, ＜1.295 mmol/L for women or treatment for dyslipidemia with lipid-lowering agent.

**3. Imaging data acquisition**

All MRI data were acquired on a 3T MRI scanner (GE DISCOVERY MR750, USA) in the Center of Information Medicine Research in University of Electronic Science and Technology of China. High-spatial-resolution T1-weighted images were acquired by using a three-dimensional fast spoiled gradient-echo sequence. The main parameters included repetition time [TR]/ echo time [TE]=6.008/1.984; flip angle [FA]=9°; field of view [FOV], 256×256 mm^2^; matrix=256×256; section thickness, 1 mm (no gap), voxel size, 1×1×1 mm^3^. Resting-state brain fMRI was collected using gradient-echo echo-planar imaging (EPI) sequences (TR=2000ms, TE=30ms, [FOV]=240×240cm^2^, [FA]=90°, matrix=64×64, slice thickness/gap=4mm/0.4mm, voxel size, 3.75×3.75×4 mm^3^). The brain fMRI scanning lasted for 510s, and a total of 255 volumes were collected. During the fMRI scanning, all participants were instructed to keep eyes open and looking at the cross-plus on the screen.

**4. Voxel-based morphometry analysis**

High-spatial-resolution T1-weighted MRI data were processed with standard voxel-based morphometry (VBM) by using the Computational Anatomy Toolbox (CAT12; <http://dbm.neuro.unijena.de/cat12/>) in statistical parametric mapping software (SPM12; http://[www.fil.ion.ucl.ac.uk/spm/](http://www.fil.ion.ucl.ac.uk/spm/)). Firstly, all structural images were checked for artifacts and reoriented to adjust image origins at the anterior commissure. Secondly, the examined images were normalized to Montreal Neurologic Institute (MNI) space reslicing to 1.5×1.5×1.5 mm^3^ voxels and segmented into gray matter, white matter, and cerebrospinal fluid areas. Thirdly, weighted average (IQR) that combines measures of noise and spatial resolution of the images was used to evaluate the weighted overall image quality of T1-weighted images. The weighted average (IQR) for all images exceeded the 75% threshold. Sample homogeneity was assessed using the “Check sample homogeneity” tool in CAT12. The correlation values for all images exceeded the 0.7 threshold. After the data quality and sample homogeneity were checked, the segmented gray matter (GM) images were smoothed by using an 8-mm full width at half maximum Gaussian kernel. Finally, the GM optimal threshold mask, created with all subjects’ GM images, was applied to eliminate non-GM voxels. The generated smoothed GM images were used as the gray matter volume (GMV) for subsequent group comparisons.

**5. Preprocessing of resting-state functional MRI**

Functional imaging data were preprocessed using DPABI (4) and NIT toolboxes (5). Data preprocessing steps were included in this study as following: (1) removing initial 5 volumes of imaging data; (2) slice-timing correction; (3) realignment; (4) normalized to the MNI space reslicing to 3×3×3 mm^3^ voxels; (5) image smoothing (FWHM 8 mm); (6) temporal filtering was performed at band-pass 0.01–0.08 Hz; (7) nuisance signals were regressed out, including six motion parameters, their first temporal derivative, and the squares of all these 12 parameters, white matter signal, cerebrospinal fluid signal. The global signal was not regressed out. All participants’ threshold whose maximum translation in any of the orthogonal directions larger than 2.5 mm or rotation larger than 2.5° were excluded from subsequent analysis.

**6. Genotyping of polymorphisms of CYP2D6 gene**

Genotyping was performed to analyze the genetic polymorphisms of following list of genes (Supplementary Methods Table1).

**Supplementary Methods Table1** The genetic polymorphisms of CYP2D6

| Gene | SNP/Allele | Gene | SNP/Allele |
| --- | --- | --- | --- |
| CYP2D6 | rs1065852 | CYP2D6 | rs28371725 |
| CYP2D6 | rs1135840 | CYP2D6 | rs5030867 |
| CYP2D6 | rs1135835 | CYP2D6 | rs16947 |

Briefly, the genomic DNA was isolated from Dried Blood Spot, and using Qubit 4 Fluorometer to accurately measure DNA quantity. The single nucleotide polymorphisms (SNPs) of interest were amplified using multiplex polymerase chain reaction (PCR) with forward and reverse primer pairs (Supplementary Methods Table2), and PCR reagents in 5ul reaction volume, containing 10 ng of genomic DNA, 0.1 μM of each oligonucleotide primer, 1x PCR buffer, 2mM dNTP and 0.2U/ul PCR Enzyme.

**Supplementary Methods Table2** Genotyping primers used in this study

| SNP | Forward primer | Reverse primer | Genotyping primer |
| --- | --- | --- | --- |
| rs1065852 | TTCTGGTAGGGGAGCCTCAG | GTGAGGCAGGTATGGGGCTA | CGCTGGGCTGCACGCTAC |
| rs28371725 | GGCCCTGACACTCCTTCTTG | GCTGACAGGTGCAGAATTGG | CCCGCCTGTACCCTT |
| rs1135840 | ACTAGGTACCCCATTCTAGC | CCATGGTGTCTTTGCTTTCC | CTTTGCTTTCCTGGTGA |
| rs5030867 | GGCCCTGACACTCCTTCTTG | GCTGACAGGTGCAGAATTGG | CCTGCTCATGATCCTAC |
| rs1135835 | CCAGGAAAGCAAAGACACCA | TCCTGCCTTTCTCAGCAGGT | ggAGCTTCTCGGTGCCC |
| rs16947 | GGCCCTGACACTCCTTCTTG | GCTGACAGGTGCAGAATTGG | ttAGAGCAGCTTCAATGATGAGAACCTG |

The PCR reactions were performed as follows: 1 cycle at 95℃ for 2 min; followed by 45 cycles at 95℃ for 30 s, 56℃ for 30 s, and 72°C for 1 min; further followed by 1 cycle at 72 C for 5 min. After amplification, the PCR products were treated with one unit each of shrimp alkaline phosphatase and exonuclease I at 37 °C for 40 min and 85°C for 5 min to purify the amplified products. The PCR products were genotyped using the iPLEX Pro Genotyping Reagent kit (Agena Bioscience; San Diego, CA, USA) and Mass ARRAY Dx Analyzer (Agena Bioscience; San Diego, CA, USA). Genotyping primers of the SNPs used in this study was list in (Additional file 1: Table S1). To assess the CYP2D6 gene copy number, we used TaqMan Copy Number Assays (assay ids: Hs00010001_cn (Ex9), and the assay was performed with an internal control - RNaseP TaqMan Copy Number Reference Assay (assay id: 4403326; Applied Biosystems, Forest City, CA, USA). 5-HTTLPR was amplified with the following primers: FWD 5′- TGTACCCCTCCTAGGATCGCTC−3′ and REV 5′- TTCTGGTGCCACCTAGACGC−3′. PCR amplification was performed in a total volume of 20 µL solutions containing the following compositions: 10 µL of 2× PCR buffer for KOD FX (TOYOBO, Osaka, Japan), 4 µL of 12 mM dNTP mix, 0.6 µL each of 10 µM primers, 2 U of KOD FX (TOYOBO) and 10 ng of genomic DNA. The thermocycling conditions were as follows: an initial cycle of 4 min at 95 °C, followed by 40 cycles of 30 s at 95 °C, 62℃ for 30 s, and 2 min at 72 °C; further followed by 1 cycle at 72 C for 7 min. All PCR amplicons were analyzed by 2% Agarose Gel Electrophoresis.

**7. Schema of calculating individual CYP2D6 activity score**

First, according to the measured single nucleotide polymorphisms (SNP), the alleles of CYP2D6, including CYP2D6*39, CYP2D6*34, CYP2D6*2, and CYP2D6*119, were defined (Supplementary Methods Figure 1a) (Pharmacogene Variation Consortium (PharmVar; <https://www.pharmvar.org/gene/CYP2D6)>) (Gaedigk A, et al., 2021). Second, combining the alleles and copy number variants (CNV) of CYP2D6 gene, diplotype of DYP2D6 were defined (Supplementary Methods Figure 1b-c). According to the expected enzyme activities reported on the database of the PharmVar, the relationships between CYP2D6 diplotype and activity score were defined (Supplementary Methods Figure 1d).

**Supplementary Methods Figure 1**. Schema of calculating individual CYP2D6 activity score.

**8. Extraction of neuroimaging features and calculation of clinical features**

In this study, based on differences from analysis of variance analysis (ANCOVA), we were prone to focus on between-group difference between SCZ-MetS and SCZ-nMetS in cross-sectional comparison. Therefore, values of GMV were extracted from the clusters that exhibited significant difference in pairwise post-hoc analysis between the SCZ-MetS and SCZ-nMetS groups. Similarly, functional connectivity (FC) values were extracted from the edges that exhibited difference in post-hoc analysis between two groups of schizophrenia patients. In longitudinal study, we extracted values of GMV and FC based on the interaction effects. These extracted values of GMV and FC as neuroimaging features were included in partial correlation analysis.

For clinical features, the remission ratio of the PANSS positive score, for example, was calculated as follows:

$$RR_{positive} = \frac{{PANSS}_{positive\_t1}-{PANSS}_{positive\_t2}}{{PANSS}_{positive\_t1}}$$

where PANSS_positive__*_t1_* and PANSS_positive__*_t2_* refer to the positive PANSS scores at *t1* and *t2*, respectively, and RR represents the remission ratio. A similar formula was used to calculate remission ratio of PANSS negative score, PANSS general score, PANSS total scores. As the method of a previous study (6), metabolic change scores were calculated by subtracting the baseline value from the endpoint value of metabolic indexes.

**9. PLS regression analysis between interaction pattern of GMV and gene expression profile**

Pharmacogenomics Knowledge Base (PharmGKB) (7) collects, curates and disseminates knowledge about clinically actionable gene-drug associations and genotype-phenotype relationships. Therefore, PharmGKB can provide clinical annotations that summarizes all of published evidence for relationships between a particular genetic variant and a specific medication or a disease. We selected a group of genes related to both “schizophrenia” and “antipsychotics” from 117,029 annotations from pharmGKB and hypothesized the expression profiles of these genes could co-localize with GMV interaction patterns of group (SCZ-MetS and SCZ-nMetS) and time (baseline and follow-up). Allen Human Brain Atlas (AHBA) microarray dataset provides spatially defined transcriptional information of the healthy human brain (8). According to the method of estimating regional gene expression for AHBA dataset in a previous study (9), a mean of all samples in a brain region was calculated to the matrix (123 regions×10,027 gene expression levels) of transcriptional level values. Based on brain-wide gene expression information from AHBA (8), we obtained expression profiles of above-mentioned a group of genes.

Partial least squares (PLS) regression was used to analysis the relationship between expression level of the group of genes and regional GMV interaction (10, 11). In PLS regression, gene expression data were used as predictor variables of reginal GMV interaction (F-values from 123 cortical regions in left hemisphere). The first component of the PLS (PLS1) was identified as the linear combination of gene expression values, which was most strongly correlated with interaction pattern of GMV. The statistical significance of the variance explained by PLS1 was tested by a permutation test based on spherical rotations of GMV interaction pattern (5000 times) to test the null hypothesis that PLS1 explained no more covariance between the GMV interaction map and expression of a group of specific genes related to both “schizophrenia” and “response to antipsychotics” than expected by chance. Variability of each gene's PLS1 was estimated via bootstrapping. The ratio of the weight of each gene to its bootstrap standard error was used to calculate the Z score and rank the genes according to their contributions to PLS1. The set of genes with an FDR (*p* < 0.05) was GMV interaction pattern gene list, including negative weights (PLS1^−^) genes and positive weights (PLS1^+^) genes.

**Supplementary Figures**

**Figure S1. The between-group difference of GMV among SCZ-MetS, SCZ-nMetS, HC-MetS and HC-nMetS based on one-way ANCOVA (unthresholded).**

**Figure S2. The post-hoc of gray matter volume among SCZ-MetS, SCZ-nMetS, HC-MetS and HC-nMetS** **based on one-way ANCOVA.** SCZ-MetS showed reduced GMV in bilateral orbital inferior frontal gyrus, bilateral orbital middle frontal gyrus, left anterior cingulum cortex and right middle cingulum cortex compared with HC-MetS or HC-nMetS. Meanwhile, SCZ-MetS showed worse GMV atrophy in these regions compared with SCZ-nMetS. Consistently, HC-MetS did not exhibit significant difference compared with HC-nMetS. (a) Left orbital inferior frontal gyrus; (b) Right orbital inferior frontal gyrus; (c) Left orbital middle frontal gyrus; (d) Right orbital middle frontal gyrus; (e) Left middle frontal gyrus; (f) Left anterior cingulum cortex; (g) Left precentral gyrus; (h) Right middle cingulum cortex.

**Figure S3. One-way ANCOVA of GMV atrophy-dependent FC at baseline (*p* < 0.05, FDR Corrected).** (a) Left map: mask of GMV atrophy in left insula. Right map: map of F-statistic values for one-way ANCOVA of left insula seed-to-voxel FC. (b) Left map: mask of GMV atrophy in right insula. Right map: map of F-statistic values for one-way ANCOVA of right insula seed-to-voxel FC. (c) Left map: mask of GMV atrophy in left caudate. Right map: map of F-statistic values for one-way ANCOVA of left caudate seed-to-voxel FC. (d) Left map: mask of GMV atrophy in right caudate. Right map: map of F-statistic values for one-way ANCOVA of right caudate seed-to-voxel FC.

**Figure S4. Repeated measures ANCOVA of GMV interaction-dependent FC during the follow-up (*p* < 0.05, GRF Corrected).** (a-f) Left map: mask of GMV with interaction. Right map: map of F-statistic values for repeated measures ANCOVA of GMV interaction-dependent seed-to-voxel FC.

**Figure S5. Group × time interaction effect of GMV interaction-dependent FC and clinical relevance in longitudinal study.** (a) Schematic diagram of interaction effect of GMV interaction-dependent FC between right insula and other regions. INS_R: right insula; MCC_L: left middle cingulum cortex; MCC_R: right middle cingulum cortex; SMA_L: left supplementary motor area; CRBL8_L: left cerebellum lobule Ⅷ; (b) Group × time interaction effect of FC between right insula and left middle cingulum cortex; (c) Group × time interaction effect of FC between right insula and right middle cingulum cortex; (d) Group × time interaction effect of FC between right insula and left cerebellum lobule Ⅷ; (e) Group × time interaction effect of FC between right insula and left supplementary motor area; (f) Correlation between change of FC between right insula and left supplementary motor area and remission rate of total score of PANSS ; Black asterisks show significance of interaction effect. Blue asterisks show significance of changed FC between baseline and follow-up in SCZ-MetS. Orange asterisks show significance of changed FC between baseline and follow-up in SCZ-nMetS. **p < 0.05, **p < 0.01, ***p < 0.001*

**Figure S6. Molecular functions, cellular components and KEGG pathway related to GMV interaction pattern.** (a) Enrichment analysis based on gene ontology (GO) molecular functions (MF) revealed top seven enriched terms (*p* < 0.05, FDR corrected). (b) Enrichment analysis based on cellular components (CC) revealed top seven enriched terms related to disease (*p* < 0.05, FDR corrected). (c) Enrichment analysis based on KEGG pathway revealed top seven enriched terms related to disease (*p* < 0.05, FDR corrected).

**Supplementary Tables**

**Supplementary Table 1**

Clinical characteristics of metabolic syndrome in patients with schizophrenia at baseline

| Characteristic | SCZ-MetS | SCZ-nMetS | Unadjusted |
| --- | --- | --- | --- |
|  | Mean (SD) | Mean (SD) | p-value |
| Body Mass Index | 25.25 (3.23) | 22.02 (3.71) | < 0.001* |
| Fast blood glucose (mmol/L) | 5.50 (1.13) | 4.9 (0.71) | 0.01* |
| Triglycerides (mmol/L) | 1.59 (0.68) | 0.99 (0.43) | < 0.001* |
| High-density lipoprotein (mmol/L) | 0.96 (0.26) | 1.23 (0.28) | < 0.001* |
| Systolic blood pressure | 117.66 (10.23) | 116.69 (12.52) | 0.739 |
| Diastolic blood pressure | 76.34 (7.47) | 76.47 (7.69) | 0.99 |

Note: we only collected 64 patients’ BMI index (SCZ-MetS, (*n* = 30), SCZ-nMetS (*n* = 34)). ** p < 0.05*;

**Supplementary Table 2**

Clinical characteristics of metabolic syndrome in healthy controls at baseline

| Characteristic | HC-MetS | HC-nMetS | Unadjusted |
| --- | --- | --- | --- |
|  | Mean (SD) | Mean (SD) | p-value |
| Body Mass Index | 29.92 (3.47) | 22.29 (2.75) | < 0.001* |
| Fast blood glucose (mmol/L) | 6.16 (1.49) | 5.51 (0.94) | 0.03* |
| Triglycerides (mmol/L) | 2.71 (2.45) | 1.19 (0.44) | < 0.001* |
| High-density lipoprotein (mmol/L) | 0.99 (0.27) | 1.37 (0.29) | < 0.001* |
| Systolic blood pressure | 133.38 (17.99) | 121.35 (14.66) | 0.004* |
| Diastolic blood pressure | 88.41 (11.08) | 81.39 (10.69) | 0.009* |

Note: ** p < 0.05*;

**Supplementary Table 3**

Demographic information and clinical characteristics of two groups of patients who completed follow-up (*n* = 33)

| Characteristic | SCZ-MetS (*n* = 17) | SCZ-nMetS (*n* = 16) | Unadjusted |
| --- | --- | --- | --- |
|  | Mean (SD) | Mean (SD) | p-value |
| Gender (male/female) | 14/3 | 14/2 | 0.68 |
| Age (years) | 48.47 (7.37) | 46.19 (9.52) | 0.45 |
| Education (years) | 11.94 (2.93) | 10.94 (2.65) | 0.31 |
| Disease duration (years) | 20.06 (9.44) | 21.81 (8.58) | 0.59 |
| Medicine dose (chlorpromazine equivalent) | 349 (159.7) | 362.8 (116.3) | 0.17 |

**Supplementary Table 4**

Clinical characteristics of psychiatric symptoms and metabolic syndrome of two groups of patients who completed follow-up

| Characteristic | SCZ-MetS (*n* = 17) | | | SCZ-nMetS (*n* = 16) | | | F | Effect size (η^2^) | P for interaction |
| --- | --- | --- | --- | --- | --- | --- | --- | --- | --- |
|  | Baseline | Follow-up | Change | Baseline | Follow-up | Change |  |  |  |
|  | Mean (SD) | Mean (SD) | Unadjusted | Mean (SD) | Mean (SD) | Unadjusted |  |  |  |
| PANSS total score | 68.2 (14.7) | 53.69 (12.8) | −10 (6) *** | 73.9 (14.9) | 68.1 (13) | −10.8(7.3) ** | 0.15 | 0.006 | 0.7 |
| PANSS positive symptoms score | 12.2 (4.6) | 9.8 (3.6) | −2.4 (2) *** | 13.9 (4.7) | 11.4 (3.7) | −2.5(2.9) *** | 0.02 | 0.001 | 0.9 |
| PANSS negative symptoms score | 24.9 (7) | 20.9 (6.3) | −4.1 (3) *** | 25.8 (6) | 21.9 (5.7) | −3.9(3.6) *** | 0.03 | 0.001 | 0.86 |
| PANSS general symptoms score | 31 (6.2) | 27.3 (5.6) | −3.8(3) *** | 34.3 (8.1) | 29.9 (6.9) | −4.4 (3.5) *** | 0.47 | 0.02 | 0.5 |
| Fast blood glucose (mmol/L) | 5.46 (1.43) | 5.8 (1.28) | −0.001 (0.7) | 4.8 (0.67) | 4.72 (0.7) | −0.1 (0.8) | 1.2 | 0.04 | 0.3 |
| Triglycerides (mmol/L) | 1.42 (0.82) | 1.4 (0.59) | −0.19 (0.9) | 1.02 (0.62) | 1 (0.5) | −0.04 (0.6) | 0.2 | 0.008 | 0.7 |
| Total cholesterol (mmol/L) | 3.69 (0.8) | 3.78 (0.86) | 0.1 (0.86) | 3.9 (0.83) | 4.2 (1.4) | 0.36 (0.87) | 0.5 | 0.019 | 0.5 |
| High-density lipoprotein (mmol/L) | 1.09 (0.21) | 1.16 (0.22) | 0.12 (0.15) | 1.2 (0.18) | 1.3 (0.31) | 0.08 (0.26) | 0.04 | 0.001 | 0.8 |
| [Low](javascript:;) [density](javascript:;) [lipoprotein](javascript:;) (mmol/L) | 1.88 (0.6) | 1.94 (0.7) | −0.004 (0.6) | 2.1 (0.57) | 2.3 (0.98) | 0.27 (0.56) | 0.8 | 0.03 | 0.4 |
| Systolic blood pressure | 119.4 (8.25) | 122.6 (9.36) | 2.8 (9.4) | 114.5 (12.6) | 116.3 (9.8) | 1.69 (13.3) | 0.2 | 0.009 | 0.7 |
| Diastolic blood pressure | 77 (6.89) | 79.33 (8.37) | 4.8 (15.1) | 73.3 (8.4) | 74.6 (7.2) | 1.23 (10.25) | 0.9 | 0.04 | 0.4 |

Note: **p < 0.05,* ***p < 0.01,* ****p < 0.001*

**Supplementary Table 5**

Moderation effect of change score of FBG on association between changed GMV of right insula and remission rate of positive symptom

| **Dependent variable** | **Independent variable** | ***R^2^*** | ***F*** | ***B*** | ***SE*** | ***T*** | ***P*** | **95%CI** |
| --- | --- | --- | --- | --- | --- | --- | --- | --- |
| Remission rate of positive symptom | Intercept | 0.17 | 6.37* | 0.79 | 0.41 | 1.91 | 0.07 | [−0.07, 1.65] |
|  | Change score of FBG |  |  | −0.02 | 0.03 | −0.64 | 0.53 | [−0.08, 0.04] |
|  | Changed GMV |  |  | 1.31 | 1.22 | 1.08 | 0.29 | [−1.21, 3.84] |
|  | Change score of FBG × Changed GMV |  |  | 4.8 | 1.9 | 2.5 | 0.019 | [0.85, 8.7] |

**p < 0.05*

**Supplementary Table 6**

Ranked weights of PLS1 genes

| PLS1^−^ genes | Z-score | PLS1+ genes | Z-score |
| --- | --- | --- | --- |
| NALCN | −9.76 | CACNA1B | 9.03 |
| CSMD1 | −9.74 | ATP1A2 | 9 |
| DTNBP1 | −9.71 | CCKBR | 7.45 |
| INSIG2 | −9.5 | ADRB2 | 7.19 |
| EIF2AK4 | −9.44 | CACNB4 | 7.14 |
| CACNA2D3 | −9.35 | CLCN6 | 6.99 |
| CNTN4 | −9.17 | C3 | 6.77 |
| NPAS3 | −9.05 | ADCY2 | 6.73 |
| LEPROT | −9.02 | CDH4 | 6.06 |
| LRP1B | −8.81 | AGT | 5.93 |
| UCP3 | −8.72 | BDNF | 5.1 |
| SLC44A3 | −8.68 | CACNB2 | 4.6 |
| SMYD3 | −8.48 |  |  |
| SLC25A14 | −8.17 |  |  |
| NUBPL | −8.04 |  |  |
| HRH3 | −7.76 |  |  |
| SV2C | −7.71 |  |  |
| CACNG3 | −7.58 |  |  |
| TMEFF2 | −7.76 |  |  |
| RABEP1 | −7.36 |  |  |
| PPA2 | −7.28 |  |  |
| CARTPT | −7.14 |  |  |
| MAML3 | −7.13 |  |  |
| NRG3 | −7.09 |  |  |
| EHF | −6.59 |  |  |
| RIMS1 | −6.44 |  |  |
| TBC1D1 | −6.38 |  |  |
| SNAP25 | −5.54 |  |  |
| PSMD14 | −4.72 |  |  |
| ABCG2 | −4.13 |  |  |
| NRXN1 | −4.07 |  |  |
| SH2B1 | −3.78 |  |  |

**References**

1.	Deurenberg-Yap M CS, Deurenberg P. Elevated body fat percentage and cardiovascular risks at low body mass index levels among Singaporean Chinese, Malays and Indians. Obes Rev. 2002.

2.	Alberti KG ZP, Shaw J. Metabolic syndrome--a new world-wide definition. A Consensus Statement from the International Diabetes Federation. Diabet Med. 2006.

3.	Lee J, Ma S, Heng D, Tan CE, Chew SK, Hughes K, et al. Should central obesity be an optional or essential component of the metabolic syndrome? Ischemic heart disease risk in the Singapore Cardiovascular Cohort Study. Diabetes Care. 2007;30(2):343-7.

4.	Yan CG, Wang XD, Zuo XN, Zang YF. DPABI: Data Processing & Analysis for (Resting-State) Brain Imaging. Neuroinformatics. 2016;14(3):339-51.

5.	Dong L, Luo C, Liu X, Jiang S, Li F, Feng H, et al. Neuroscience Information Toolbox: An Open Source Toolbox for EEG-fMRI Multimodal Fusion Analysis. Front Neuroinform. 2018;12:56.

6.	Luckhoff H, Phahladira L, Scheffler F, Asmal L, du Plessis S, Chiliza B, et al. Weight gain and metabolic change as predictors of symptom improvement in first-episode schizophrenia spectrum disorder patients treated over 12months. Schizophr Res. 2019;206:171-6.

7.	Klein TE, Chang JT, Cho MK, Easton KL, Fergerson R, Hewett M, et al. Integrating genotype and phenotype information: an overview of the PharmGKB project. Pharmacogenetics Research Network and Knowledge Base. Pharmacogenomics J 2001.

8.	Hawrylycz MJ, Lein ES, Guillozet-Bongaarts AL, Shen EH, Ng L, Miller JA, et al. An anatomically comprehensive atlas of the adult human brain transcriptome. Nature. 2012;489(7416):391-9.

9.	Arnatkeviciute A, Fulcher BD, Fornito A. A practical guide to linking brain-wide gene expression and neuroimaging data. Neuroimage. 2019;189:353-67.

10.	Li J, Seidlitz J, Suckling J, Fan F, Ji GJ, Meng Y, et al. Cortical structural differences in major depressive disorder correlate with cell type-specific transcriptional signatures. Nat Commun. 2021;12(1):1647.

11.	Morgan SE, Seidlitz J, Whitaker KJ, Romero-Garcia R, Clifton NE, Scarpazza C, et al. Cortical patterning of abnormal morphometric similarity in psychosis is associated with brain expression of schizophrenia-related genes. Proc Natl Acad Sci U S A. 2019;116(19):9604-9.
